# Fabrication of Activated Carbon Fibers with Sheath-Core, Hollow, or Porous Structures via Conjugated Melt Spinning of Polyethylene Precursor

**DOI:** 10.3390/polym12122895

**Published:** 2020-12-03

**Authors:** Jong Sung Won, Ha Ram Lee, Min Jun Lee, Min Hong Jeon, Seung Goo Lee, Yong Lak Joo

**Affiliations:** 1 Robert Frederick Smith School of Chemical and Biomolecular Engineering, Cornell University, Ithaca, NY 14853, USA; jw2636@cornell.edu; 2 Department of Advanced Organic Materials & Textile Engineering, Chungnam National University, Daejeon 34134, Korea; haram523@cnu.ac.kr (H.R.L.); irai94@o.cnu.ac.kr (M.J.L.); hong831@o.cnu.ac.kr (M.H.J.); lsgoo@cnu.ac.kr (S.G.L.)

**Keywords:** activated carbon fiber, polyethylene precursor, hollow carbon fiber, sheath-core activated carbon fiber

## Abstract

Using polyethylene as carbon precursor, we have fabricated cost-effective carbon fibers with a sheath-core structure via conjugate melt spinning. Low-density polyethylene (LDPE) and high-density polyethylene (HDPE) were used as the sheath and core of the fiber, respectively, while sulfonation with sulfuric acid was conducted to enable the crosslinking of polyethylene. We demonstrated that carbonization and activation of the sheath-core-structured polyethylene fiber can result in a well-developed microporous structure in the sheath layer, and due to the core-sheath structure, the resulting activated carbon fibers exhibit a high tensile strength of ~455 MPa, initial modulus of ~14.4 GPa, and Brunauer-Emmett-Teller (BET) surface area of ~1224 m^2^/g. Finally, activated carbon fibers with a hollow, sheath-core, and porous were successfully fabricated by controlling the degree of crosslinking of the LDPE/HDPE sheath-core fiber.

## 1. Introduction

Activated carbon fiber is an important nano-porous carbon material used in filter applications because of its excellent adsorption properties. It has an intrinsic porous structure with low meso porosity and no macro porosity. Therefore, it has also been employed for heavy metal removal, as an adsorbent for hazardous volatiles, and for vapor sensing and gas storage [1,2,3,4,5]. The production of activated carbon fiber is not very different from that for carbon fiber. To date, many kinds of activated carbon fibers have been developed using carbon precursor fibers, such as polyacrylonitrile (PAN), pitch, rayon, and phenolic. These activated carbon fibers exhibit proper nano-porous structures and have been used for filtering hazardous volatile gases and very fine dusts-pollutants which have recently attracted global attention due to air pollution [6,7,8,9,10,11].

However, the typical cost of activated carbon fiber is much higher than that of other microporous materials, because of the cost of the precursor material and manufacturing process. This limit widespread usage of carbon fiber and activated carbon fibers. Therefore, recently, many efforts have focused on developing low-cost carbon fiber using low-cost precursor materials. One promising precursor material is polyethylene, which has the advantages of a very low cost and easy processing via conventional melt spinning [12,13,14,15,16,17]. 

Recently, some studies have reported successful manufacturing of carbon fiber from a polyethylene precursor. To prepare carbon fiber using a polyethylene precursor, sulfonation with sulfuric acid is typically performed to stabilize the precursor. This results in slightly poorer mechanical properties compared to conventional carbon fibers. Nevertheless, it provides novel routes for carbon fiber processing with a low-cost precursor. However, there have been almost no studies on developing activated carbon fiber using a polyethylene precursor. A polyethylene-based activated carbon fiber would be an important absorbent material with low cost and versatility [18,19,20,21,22,23,24,25].

On the other hand, the poor mechanical properties of most activated carbon fibers with a microporous structure make it very difficult to handle or treat for various applications. Lack of mechanical strength limits their usage in an unloaded state. Therefore, the fibers cannot be easily applied for a fabric structure which requires extensional properties and mechanical endurance [26,27].

In the current study, we fabricated activated carbon fiber using polyethylene fiber via conjugate spinning. Thus, a sheath-core structure of the precursor fiber was obtained, yielding better mechanical properties than those of conventional monoaxial activated carbon fibers. Two types of polyethylene, high-density (HDPE) and low-density (LDPE), were used as the core and sheath materials, respectively. HDPE in the core can maintain the mechanical strength of the fiber, while LDPE, with its low compactness in the sheath, can be activated easily. This unique bilateral fiber structure makes it possible to obtain a fully activated sheath layer and less-activated core layer with proper mechanical strength. In this study, the effects of the draw ratio of the precursor fiber on the sulfonation based stabilization process were investigated. After carbonization and activation of the polyethylene fiber, the nano-porous structure with a high pore area in resulting carbon fibers was analyzed. Finally, the mechanical properties of the prepared activated carbon fiber were tested to evaluate the effectiveness of the sheath-core fiber structure.

## 2. Materials and Methods 

### 2.1. Materials

LDPE (XJ700, Lotte Chemical Co., Daejeon, Korea) was chosen as the sheath component and HDPE (2600F, Lotte Chemical Co., Daejeon, Korea) was used for the core of the sheath-core fibers. 

### 2.2. Preparation of Sheath-Core Fibers

**Figure 1:**
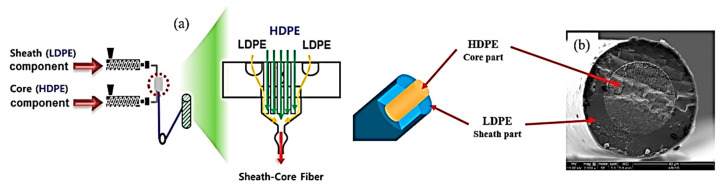
(**a**) Schematic melt spinning of sheath-core fiber and (**b**) SEM image of LDPE/HDPE sheath-core fiber manufactured by a conjugate melt-spinning.

LDPE/HDPE sheath-core fibers were prepared by conjugate melt-spinning at 205 °C at a take-up speed of 850 m/min, as shown schematically in Figure 1a. The content ratio of LDPE/HDPE in the sheath-core fiber was set to 50:50 by weight percent, as seen the scanning electron microscopy (SEM) image, Figure 1b. The drawing treatment of LDPE/HDPE sheath-core fibers was performed using a self-made drawing machine, at draw ratios of 1.2, 1.3, 1.5, and 1.6 at 95 °C.

### 2.3. Cross-Linking, Carbonization, and Activation of Sheath-Core Fibers

**Figure 2:**
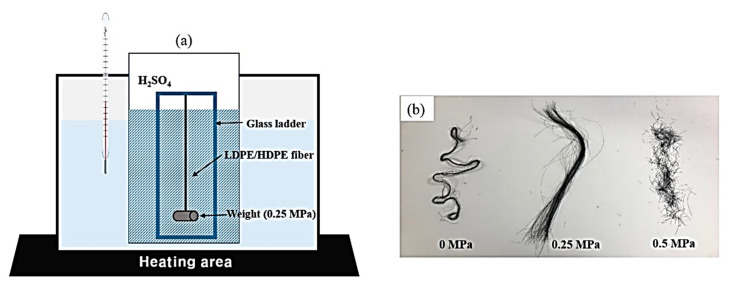
(**a**) Concept illustration of the crosslinking process and (**b**) images of the sulfated fibers under loads of 0, 0.25 and 0.5 MPa for the precursor fibers.

The obtained precursors were stabilized through a dipping treatment in sulfuric acid (H_2_SO_4_, 95%, Samchun Co., Seoul, Korea) as shown in Figure 2a. Figure 2b shows the shape of the sulfated fibers under loads of 0, 0.25 and 0.5 MPa. When a load of 0.5 MPa was applied, it was confirmed that the fiber was cut. Therefore, in this study, sulfuric acid treatment was carried out with a load of 0.25 MPa. Uniaxially aligned LDPE/HDPE sheath-core fibers were stabilized by dipping, with a load of 0.25 MPa applied at different temperatures and times, as shown in Table 1. Carbonization was performed in nitrogen atmosphere at a final temperature of 900 °C and a heating rate of 5 °C/min. The process time at the maximum temperature was set as 5 min to complete carbonization. After completion of carbonization, the furnace was turned off and maintained in nitrogen atmosphere. This condition of inert atmosphere was maintained until the temperature inside the furnace reactor reached room temperature. Activated sheath-core carbon fibers were prepared by chemical activation. Activation of the cross-linked LDPE/HDPE sheath-core fibers was carried out by dipping for 24 h using a 1–4 M KOH solution and drying for 12 h in a convection oven at 100 °C.

### 2.4. Characterization of Sheath-Core Fibers

The thermal transition behavior of the LDPE/HDPE sheath-core fibers was measured by differential scanning calorimetry (DSC, Mettler-Toledo DSC1) in the temperature range of 25–250 °C at a heating rate of 10 °C/min in nitrogen atmosphere. The melting enthalpy was measured and the aromatization index (AI) was calculated to identify the level of cross-linking, using the following Equation (1) [18,28]:

(1) $$\mathrm{Aromatization}\;\mathrm{index}\;(\%)\;=\;\left(\frac{\Delta H_{0}}{\Delta H/\Delta H_{0}}\right)\;\times \;100$$

where Δ*H*_0_ is the melting enthalpy of the pristine sample and Δ*H* is the melting enthalpy of the cross-linked sample. 

The effects of the temperature characteristics of the cross-linking processes were investigated using thermogravimetric analysis (TGA, Mettler-Toledo TGA/DSC1). In TGA, all cross-linked samples were heated from 25 °C to 800 °C at a heating rate of 10 °C/min in a nitrogen atmosphere.

The surface and cross-sectional morphological features of the LDPE/HDPE sheath-core fibers after cross-linking and carbonization were characterized by SEM (S4700, HITACHI, Daejeon, Korea). To obtain cross-sectional SEM images, the samples were immersed in a liquid nitrogen bath for 5 min and then sectioned using a diamond cutter.

A Fourier transform infrared (FT-IR) spectrophotometer (Bruker Optic GmbH, ALPHA-P, Daejeon, Korea) equipped with an attenuated total reflectance (ATR) accessory was used to examine the surface composition changes in the fibers attributed to cross-linking treatment. The spectra were recorded in the transmission mode in the range of 3500–500 cm^−1^, with a spectral resolution of 4 cm^−1^, and accumulation of 128 scans for a high signal-to-noise ratio.

The tensile mechanical tests were carried out using a universal tensile testing machine (Model 4467, Instron) at a crosshead speed of 1 mm/min for the cross-linked sheath-core fibers and carbon fibers (ASTM D3822). 

The Brunauer–Emmett–Teller (BET) method was applied to determine the total surface area, while the pore size distribution and micropore volume were estimated by applying the Barret–Joyner–Halenda (BJH) method and the non-local density functional theory (NLDFT) over the adsorption isotherm. 

## 3. Results and Discussion

### 3.1. Characterization of LDPE/HDPE Sheath-Core Fibers 

**Figure 3:**
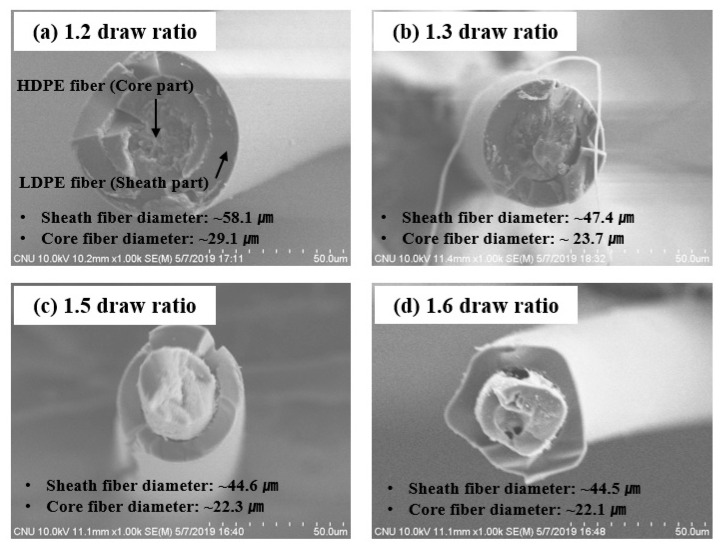
Cross-sectional SEM image of LDPE/HDPE sheath-core fibers manufactured at different draw ratio of 1.2–1.6 times.

Figure 3 shows the cross-sectional SEM images of the LDPE/HDPE sheath-core fibers prepared at different draw ratios. It is observed that the diameters of the sheath fibers as well as core fibers decrease with increasing draw ratio. We note that the interface between HDPE core and LDPE shear is smooth and compact, and the LDPE sheath is well packed in the sheath-core fibers manufactured at a draw ratio of 1.2–1.5, whereas the interface becomes less uniform in those manufactured at the higher draw ratio of 1.6, which may be attributed to the interfacial instability at high drawing speeds. Nonetheless, the ratio of core radius to shear layer thickness remains the same at about 0.5 for all draw ratios studied.

**Figure 4:**
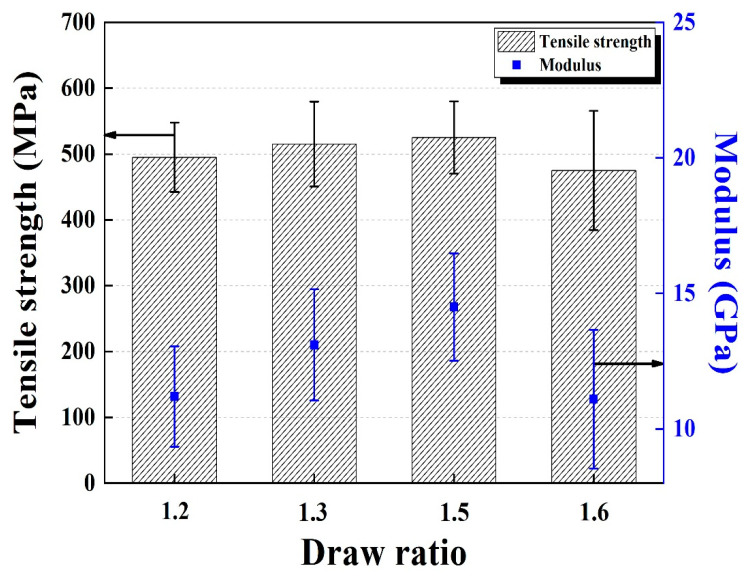
Tensile strength and initial modulus of LDPE/HDPE sheath-core fibers manufactured at different at draw ratio of 1.2–1.6 times.

Next, the tensile mechanical properties of the LDPE/HDPE sheath-core fibers manufactured at different draw ratios were investigated. The tensile strength and initial modulus of the fibers increases with increasing the draw ratio up to 1.5, as shown in Figure 4. Beyond a ratio of 1.5, however, both tensile strength and modulus decrease, possibly due to the less uniform core and sheath morphology and less circular cross section of the fibers. Therefore, LDPE/HDPE sheath-core fibers manufactured at the draw ratio of 1.5 were used to obtain the activated carbon fibers.

### 3.2. Characterization of Cross-Linked LDPE/HDPE Sheath-Core Fibers 

**Figure 5:**
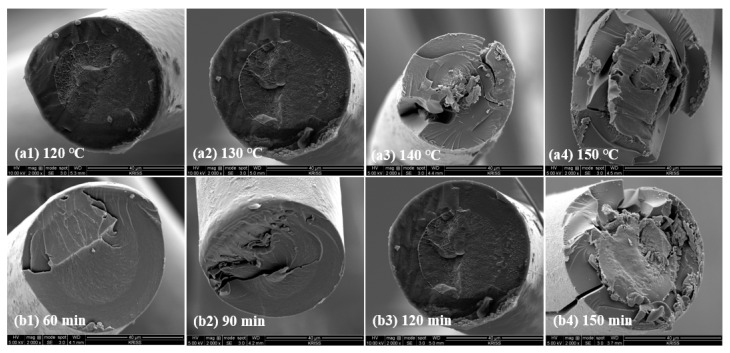
SEM images of the sulfonated LDPE/HDPE sheath-core fibers at various crosslinking temperature and time: (**a1**–**a4**) temperatures of 120–150 °C under 0.25 MPa for 120 min; (**b1**–**b4**) duration times of 60–150 min under 0.25 MPa at 130 °C.

The LDPE/HDPE sheath-core fibers were cross-linked by sulfuric acid. Figure 5 shows the SEM images of the cross-section of the cross-linked LDPE/HDPE sheath-core fibers manufactured at different cross-linking temperatures (120–150 °C) and time (60–150 min). The range of cross-linking temperature was determined based on the melting behavior of two components: the melting transition temperature (Tm) of the LDPE sheath component in the sheath-core fibers was in the range of 96–110 °C, which was lower than that (123–133 °C) of the HDPE core component. For the cross-linked sheath-core fibers manufactured at cross-linking temperatures of 120 and 130 °C under a constant load of 0.25 MPa for 120 min (Figure 5(a1,a2)), good interaction among the sheath-core fibers as well as good structure of the sheath-core component was achieved. However, the LDPE sheath part melts at 140–150 °C, to which the core exhibit defects in the final sheath-core fiber (Figure 5(a3,a4)). For cross-linked sheath-core fibers manufactured at different cross-linking durations of 60–150 min under a constant cross-linking temperature of 130 °C and load of 0.25 MPa (Figure 5(b1–b4)), good interaction and shape of the sheath-core fibers are attained below 120 min, whereas the structure of the LDPE sheath part exhibits visible fractures above 150 min (Figure 5(b4)).

**Figure 6:**
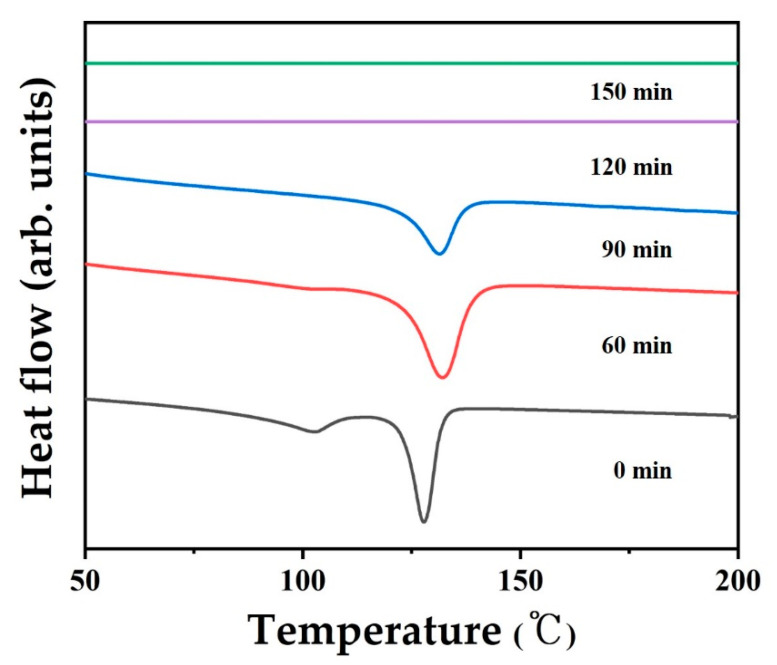
DSC patterns of the sulfonated fibers for various crosslinking time at 130 °C.

Figure 6 shows the DSC heating thermograms of the LDPE/HDPE sheath-core fibers cross-linked for 60–150 min under constant pressure and temperature of 0.25 MPa and 130 °C, respectively. In the first DSC heating curves at the bottom, double-melting endotherms are observed for the untreated LDPE/HDPE sheath-core fiber. The lower endotherm at ~102 °C is associated with the melting of the sheath-part LDPE, whereas the higher endotherm at ~128 °C is associated with the melting of the core-part HDPE. For the sheath-core fiber cross-linked for greater than 60 min, only a single endothermic peak appears at ~130 °C, because the cross-linking of LDPE has completed. The lower melting enthalpy for the sheath-core fiber cross-linked for 150 min is attributed to the increased cross-linking of the HDPE core part in the LDPE/HDPE sheath-core fiber, compared to that cross-linked for 60 min. This indicates that the LDPE/HDPE sheath-core fiber is rendered infusible by the sulfuric acid treatment.

Table 2 lists the aromatization index (AI) values calculated from the melting enthalpy experiments. During the acid treatment at 130 °C, the melting enthalpy of core-part HDPE decreased from 215.6 J/g to 120.9, 69.1 and 0 J/g at a cross-linking time of 60, 90, 120, and 150 min, respectively, corresponding to AI values of 43.9%, 67.95% and 100%. The melting enthalpy of sheath-part LDPE decreased from 48.5 J/g to 3.9, and 0 J/g at a cross-linking time of 60, 90, 120, and 150 min, respectively, corresponding to AI values of 91.88% and 100%. The LDPE sheath part becomes completely infusible after 60 min, while the HDPE core part becomes infusible at an AI of 100% at 120 min. Therefore, from this result, it was confirmed that the crosslinking of the HDPE core part required a longer sulfonation time than the LDPE sheath part. As demonstrated in the later section, this difference in crosslinking behavior by sulfonation of LDPE and HDPE can be utilized in fabricating hollow porous carbon fibers.

**Figure 7:**
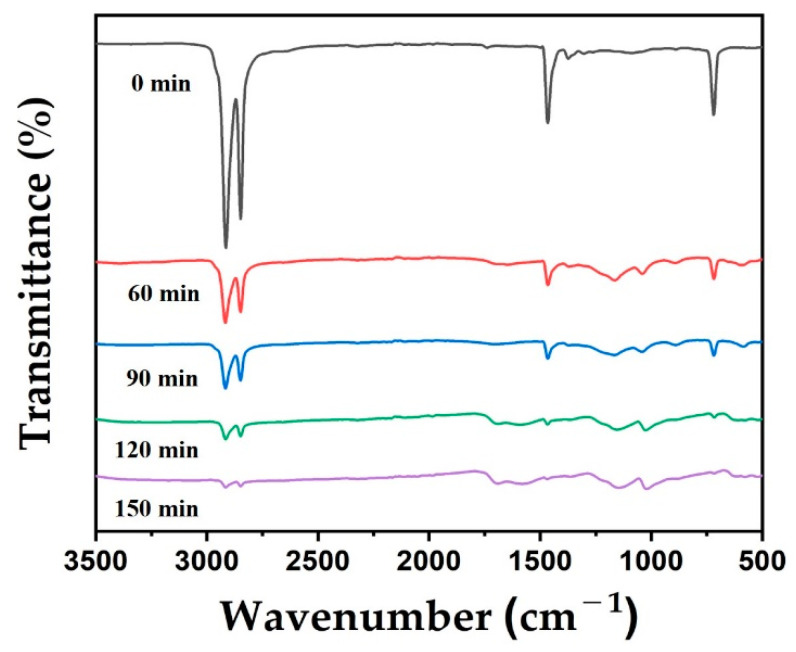
FTIR patterns of the sulfonated LDPE/HDPE sheath-core fibers for various crosslinking time at 130 °C.

Next, FTIR was used to characterize the changes of chemical structure of the LDPE/HDPE sheath-core fibers during cross-linking. Figure 7 shows the FTIR spectra of the LDPE/HDPE sheath-core fibers cross-linked for 60–150 min at 130 °C. Bands at 3000–2850 cm^−1^, 1384–1475 cm^−1^, and 710–750 cm^−1^ appear in all samples, and can be attributed to the stretching of the C–H bond in methylene groups and the C–H long-chain bond in LDPE and HDPE, respectively. With increasing duration of cross-linking, the peak for the –CH_2_ bond at 1465 cm^−1^ shifts to 1280–1080 cm^−1^ and 1040 cm^−1^, respectively. The peak intensities near 724 cm^−1^ (C–H stretching vibrations) was prominent in the pure LDPE/HDPE sheath-core structure. In succession, these were found to reduction with increasing sulfonation time. The peak intensities diminished evidently with enhanced duration and sulfonate group incorporation in the sulfonated LDPE/HDPE sheath-core fibers. This shows that sulfonation was achieved by O=S=O stretching vibrations. Therefore, the H-branch on the LDPE and HDPE chain was substituted for the sulfonic acid group of –HSO_3_ and bound to another H-branch to form the sulfone of SO_2_. In addition, with increasing duration of cross-linking, the composition of carbon and hydrogen decreased while the composition of oxygen and sulfur increased. The decrease in composition of carbon and hydrogen indicates a decrease in the number of C–H bonds, whereas the increase in the absorption for a sulfone group (SO_2_) refers to an increase in the composition of oxygen and sulfur (see Supplementary Table S1).

**Figure 8:**
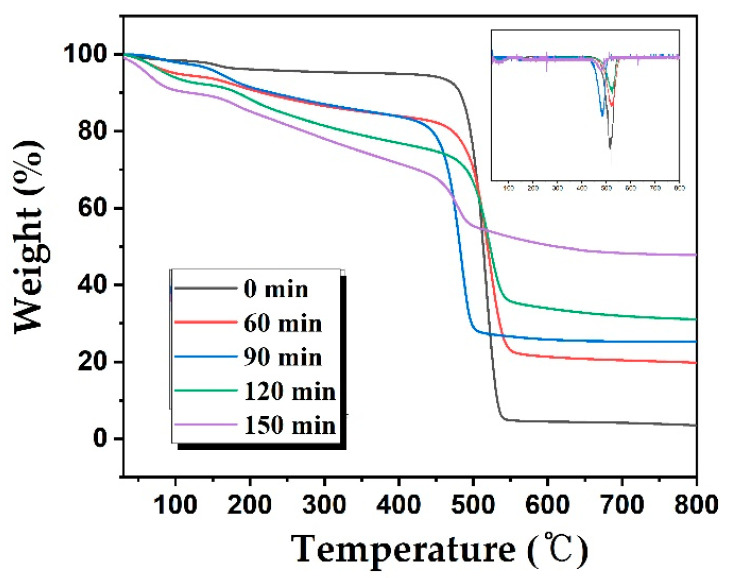
TGA results of the sulfonated LDPE/HDPE sheath-core fibers for various crosslinking times at 130 °C. The inset is corresponding differential curves for various crosslinking times at 130 °C.

Figure 8 shows the increasing weight retention with increasing cross-linking duration. TGA curves indicate that the decomposition temperatures for all LDPE/HDPE sheath-core fibers cross-linked via sulfuric acid lie in the range of 490–550 °C. The thermal stability, based on the polymer decomposition temperature and integral procedural decomposition temperature, as well as the carbonization yield increases with increasing cross-linking duration. The cross-linking reaction improves the decomposition stability and heat exhaustion, resulting from the most densely cross-linked structure at 150 min. This also enhances gel structures in the LDPE/HDPE sheath-core fibers, resulting in high carbonization yields. The LDPE/HDPE sheath-core fibers cross-linked at 150 min exhibit the highest weight retention (47.72%) among all the fibers. In addition, the flammability of the stabilized samples was determined by a separate burning test that samples were put on flame from a blow torch. The samples that do not burn in general can be carbonized without burning at higher temperature. As shown in burning test images in Supplementary Figure S1, the LDPE/HDPE sheath-core fibers cross-linked at 120–150 min did not burn, whereas the LDPE/HDPE sheath-core fibers cross-linked at 0–90 min burned in the flame. This also confirms that after 120 min of sulfonation, the LDPE/HDPE sheath-core fibers were effectively cross-linked.

**Figure 9:**
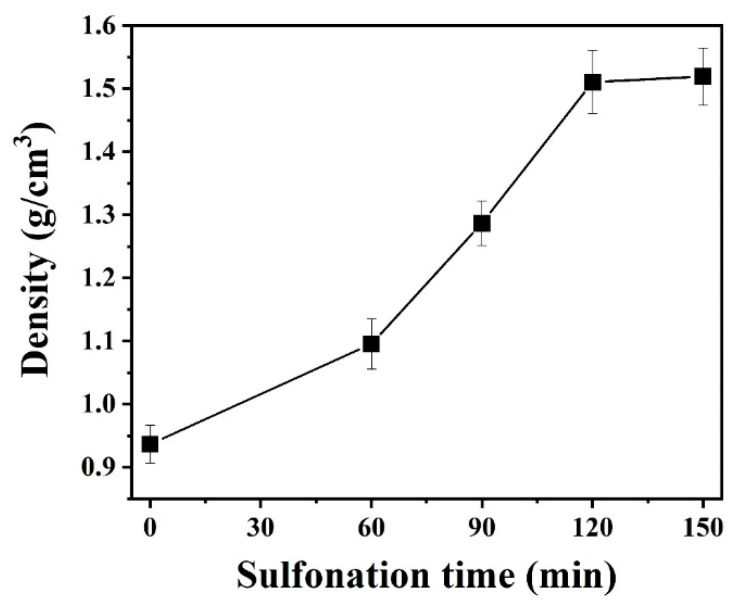
Density of the sulfonated LDPE/HDPE sheath-core fibers for various crosslinking times at 130 °C.

Figure 9 shows the change in density of LDPE/HDPE sheath-core fibers for various cross-linking times at 130 °C. It is observed that an increase in cross-linking time resulted in an increase in density from 0.9365 to 1.5191 g/cm^3^ for LDPE/HDPE sheath-core fibers until cross-linking duration of 120 min.

### 3.3. Characterization of Carbonized and Activated LDPE/HDPE Sheath-Core Fibers 

**Figure 10:**
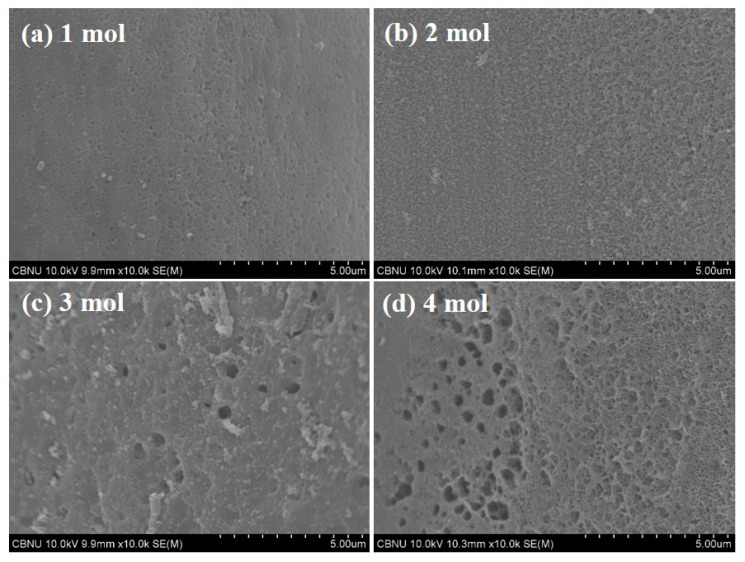
SEM images of the carbonized and activated LDPE/HDPE sheath-core fibers at various KOH molarity: (**a**) 1 mol; (**b**) 2 mol; (**c**) 3 mol; (**d**) 4 mol.

The external pore structures of the carbonized and activated LDPE/HDPE sheath-core carbon fibers are presented via SEM images in Figure 10. It is observed that the morphological features of KOH-activated LDPE/HDPE sheath-core carbon fibers are strongly influenced by KOH concentration (Figure 10a–d). The structure and distribution of pores are very non-uniform possibly due to the fast reaction of KOH. SEM images of the carbonized LDPE/HDPE sheath-core fibers at 1 molarity also indicate the presence of relatively smooth surface and few pores (Figure 10a). On the other hand, the activated LDPE/HDPE sheath-core carbon fibers at 2–4 molarity exhibit surfaces containing macro, meso, micro pores, with increased macro pores at high KOH concentration. As the KOH concentration increases, highly cracked and collapsed surfaces are obtained, indicating the violent reaction between KOH and surface carbon with increasing KOH amount. This demonstrates that proper KOH treatment can lead to the creation of micro pores and high surface areas.

**Figure 11:**
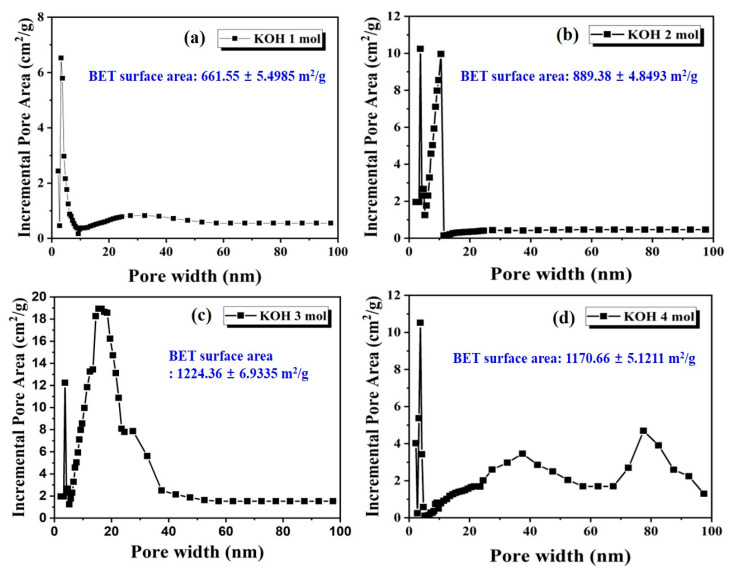
BJH analysis of nitrogen physisorption showing the incremental pore area distribution of activated LDPE/HDPE sheath-core carbon fibers at various KOH molarity: (**a**) 1 mol; (**b**) 2 mol; (**c**) 3 mol; (**d**) 4 mol.

To quantify the effect of KOH on the pore structure and surface area, we present the BJH incremental pore area distributions of the activated LDPE/HDPE sheath-core carbon fibers at four different KOH concentration in Figure 11. The nitrogen isotherm for the activated LDPE/HDPE sheath-core carbon fibers provided important information on the specific surface area and pore. It is observed that KOH chemical activation of the carbon fiber increases the surface area and porosity. The activated LDPE/HDPE sheath-core carbon fibers at 3–4 molarity exhibit better properties, with a BET surface area of 1224.36 m^2^/g and 1170.66 m^2^/g, respectively, compared with those at 1–2 molarity (661.55 m^2^/g and 889.38 m^2^/g, respectively). Thus, these results indicate that these carbon fibers activated by KOH are predominantly populated with micro pores. However, it was confirmed that when the molar ratio of KOH activation is very high, not only micro pores but also meso and macro pores are formed. Surface area analysis by BET show that the carbon fibers activated with 1 molarity to 4 molarity KOH had various surface properties in terms of the surface area and pores.

**Figure 12:**
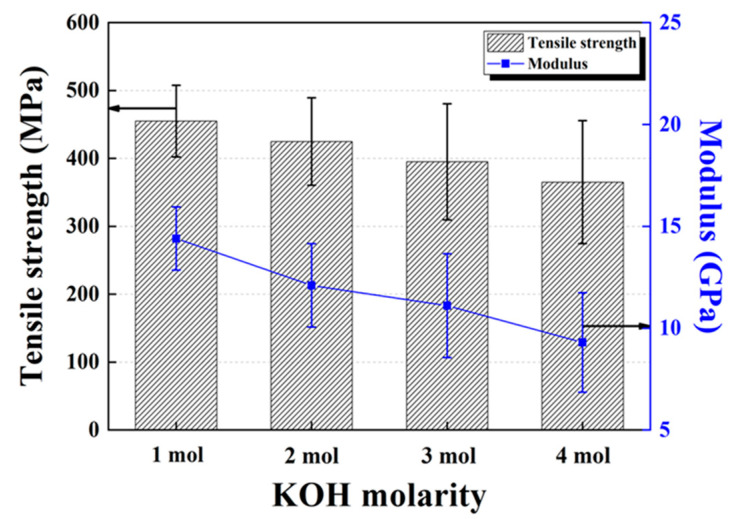
Tensile strength and initial modulus of activated LDPE/HDPE sheath-core carbon fibers at various KOH molarity.

Results for the tensile mechanical tests of the LDPE/HDPE sheath-core carbon fibers after activation are presented in Figure 12. Generally, tensile strength increases by carbonization and decreases after activation. The decrease in tensile strength with activation exhibits different strengths depending on the pore size and shape formed on the fiber surface. However, the strength of 455 MPa obtained by conjugate spinning of the sheath-core structure in the current study is regarded to be significantly higher than a single polyethylene based activated carbon fiber [29]. This improvement in tensile strength after carbonization and activation can be attributed to the bicomponent structure of the fiber, with a HDPE core and LDPE sheath, compared to conventional single-structured carbon fiber. This unique bilateral fiber structure enables the full activation of the sheath layer with lesser activation of the core layer, to provide suitable mechanical strength.

**Figure 13:**
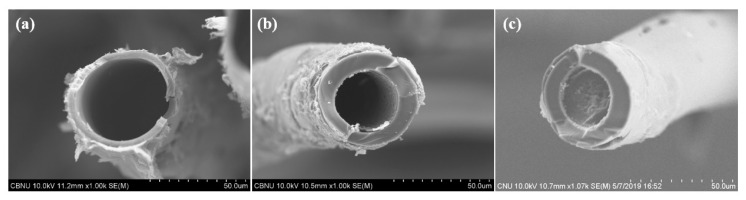
Cross section SEM images after carbonization and activation of the sulfonated LDPE/HDPE sheath-core fibers for time of 60–120 min under 0.25 MPa at 130 °C: (**a**) 60 min; (**b**) 90 min; (**c**) 120 min.

Finally, Figure 13 shows cross section SEM images after carbonization and activation of the sulfonated LDPE/HDPE sheath-core fibers for time of 60–120 min under 0.25 MPa at 130 °C. As a result, cross sections of activated carbon fibers having three different structures were confirmed. It should be noted that for the LDPE/HDPE sheath-core carbon fibers sulfonated at cross-linking time of 60 min (Figure 13a), a cross section of the activated carbon fiber having a large hollow structure was observed. The difference in cross-linking in HDPE core and LDPE sheath and thus incomplete cross-linking of the HDPE core and inner region of the LDPE sheath went through thermal decomposition during the carbonization process to form a large pore structure. On the other hand, as the sulfonation time increased to 90 min, the hollow structure became smaller due to the enhanced degree of cross-linking of the entire LDPE sheath while cross-linking of the core HDPE is still incomplete. In the case of activated sheath-core carbon fibers with 100% crosslinking of both LDPE sheath and HDPE core for 120 min sulfonation, a hollow structure was not observed as shown in Figure 13c, and the coherent core structure that could contribute to the improvement of tensile properties was observed.

## 4. Conclusions

In summary, activated LDPE/HDPE sheath-core carbon fibers were manufactured by carbonization and activation with a KOH solution after cross-linking via sulfonation at various temperatures (120–150 °C) and durations (60–150 min) under a load of 0.25 MPa. LDPE/HDPE (50/50 wt.%/wt.%) sheath-core fibers were fabricated via melt-conjugate spinning at different draw ratios from 1.2 to 1.6. The activated carbon fibers with the sheath-core structure spun at a draw ratio of 1.5 were characterized by considering the orientation, cross-linking, carbonization, and activation of the LDPE/HDPE sheath-core fibers. Consequently, the tensile properties and specific surface area of the activated LDPE/HDPE sheath-core carbon fibers were found to be maximized when cross-linking was carried out at 130 °C and 0.25 MPa for 120 min. At this condition, a small pore size, high BET surface area, high AI, and high carbonized yield were achieved, as confirmed by SEM, FTIR, DSC, TGA, and BET analyses. In addition, the tensile properties of the activated carbon fibers were improved by the presence of HDPE in the core, which eventually contributed to enhancing the final mechanical properties of the activated LDPE/HDPE sheath-core carbon fibers. Overall, the activated carbon fibers exhibited a high tensile strength of ~455 MPa, initial modulus of ~14.4 GPa, and BET surface area of ~1224.36 m^2^/g. The resulting LDPE/HDPE sheath-core activated carbon fibers could be utilized for various applications such as environmental (air purification filter, water treatment filter), chemical (recovery of organic solvents and compounds), military defense (gas masks and fire-resistant clothing), and next-generation battery materials.

## Figures and Tables

**Table 1:** Sulfuric acid treatment conditions of LDPE/HDPE sheath-core fiber.

Temperature (°C)	120, 130, 140, 150
Time (Min)	60, 90, 120, 150
Loading (MPa)	0.25

**Table 2:** Aromatization index values of LDPE/HDPE sheath-core fibers for various crosslinking time at 130 °C.

Crosslinking Time (min)		0	60	90	120	150
**Crosslink percentage (%)**	**HDPE**	0	43.9	67.95	100	100
	**LDPE**	0	91.88	100	100	100

## Supplementary Materials

The following are available online at https://www.mdpi.com/2073-4360/12/12/2895/s1, Figure S1: Burning test images of the sulfonated LDPE/HDPE sheath-core fibers for time of 60–120 min under 0.25 MPa at 130 °C.; Table S1: Elementary composition of the LDPE/HDPE sheath-core fibers for various crosslinking times at 130 °C.
