# Supplementary material for: Fabrication of Activated Carbon Fibers with Sheath-Core, Hollow, or Porous Structures via Conjugated Melt Spinning of Polyethylene Precursor
Source: Polymers (Basel). 2020 Dec 3;12(12):2895. doi: 10.3390/polym12122895 (PMC7761741; doi:10.3390/polym12122895)
Supplement: Supplementary file 1 [file polymers-12-02895-s001.pdf]

Supporting Information

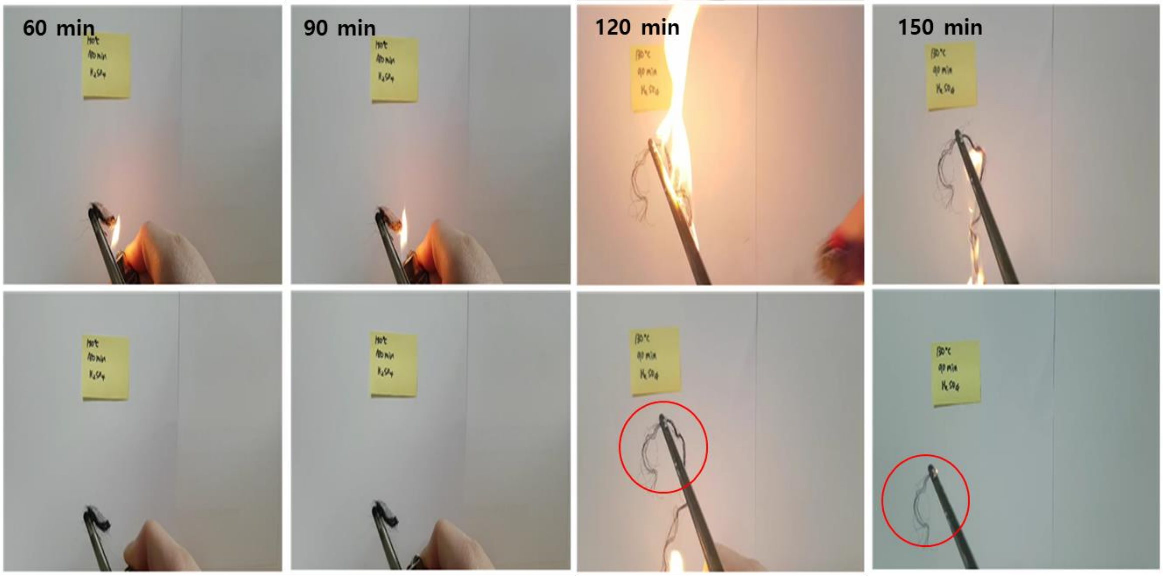

Figure S1. Burning test images of the sulfonated LDPE/HDPE sheath-core fibers for time of 60-120 min under 0.25 MPa at 130 °C.

Table S1. Elementary composition of the LDPE/HDPE sheath-core fibers for various crosslinking times at 130 ℃.

Crosslinking Time (min)	Carbon (%)	Hydrogen (%)	Oxygen (%)	Sulfur (%)
0	83.83	14.81	-	-
60	72.12	12.29	12.11	2.48
90	62.12	8.89	13.28	4.62
120	53.43	6.07	17.14	5.3
150	43.27	4.41	19.37	5.47
